# Col11a1 Regulates Bone Microarchitecture during Embryonic Development

**DOI:** 10.3390/jdb3040158

**Published:** 2015-12-16

**Authors:** Anthony Hafez, Ryan Squires, Amber Pedracini, Alark Joshi, Robert E. Seegmiller, Julia Thom Oxford

**Affiliations:** 1 Biomolecular Research Center, Boise State University, 1910 University Drive, Boise, ID 83706-1511, USA; 2 Department of Physiology and Developmental Biology, Brigham Young University, Provo, UT 84602, USA; 3 College of Dental Medicine, Roseman University, South Jordan, UT 84095, USA

**Keywords:** Collagen XI, Col11a1, mineralization, chondrodysplasia, X-ray microtomography

## Abstract

Collagen XI alpha 1 (Col11a1) is an extracellular matrix molecule required for embryonic development with a role in both nucleating the formation of fibrils and regulating the diameter of heterotypic fibrils during collagen fibrillar assembly. Although found in many different tissues throughout the vertebrate body, Col11a1 plays an essential role in endochondral ossification. To further understand the function of Col11a1 in the process of bone formation, we compared skeletal mineralization in wild-type (WT) mice and Col11a1-deficient mice using X-ray microtomography (micro-CT) and histology. Changes in trabecular bone microstructure were observed and are presented here. Additionally, changes to the periosteal bone collar of developing long bones were observed and resulted in an increase in thickness in the case of Col11a1-deficient mice compared to WT littermates. Vertebral bodies were incompletely formed in the absence of Col11a1. The data demonstrate that Col11a1 depletion results in alteration to newly-formed bone and is consistent with a role for Col11a1 in mineralization. These findings indicate that expression of Col11a1 in the growth plate and perichondrium is essential for trabecular bone and bone collar formation during endochondral ossification. The observed changes to mineralized tissues further define the function of Col11a1.

## 1. Introduction

The skeleton forms by a combination of endochondral and intramembranous ossification. Fetal long bone formation proceeds by the process of endochondral ossification in which mesenchymal stem cells condense into an anlagen, or cartilage model, then subsequently undergo chondrogenesis. Chondrocytes secrete a cartilage-specific extracellular matrix and undergo longitudinal proliferation resulting in the elongation of long bones. Undifferentiated mesenchymal cells peripheral to the cartilage anlagen develop directly into the bony collar through the process of intramembranous bone formation that does not transition through a cartilage intermediate.

Chondrocytes at the diaphysis of the developing long bone undergo further maturation and hypertrophy, followed by an exit from the cell cycle [1,2]. Hypertrophic chondrocytes expressing collagen type X, alkaline phosphatase, Runx2, osteopontin, and osteocalcin stimulate the calcification of cartilage in the hypertrophic zone of the growth plate [3,4]. Ossification begins with invasion of the calcified hypertrophic cartilage by capillaries from the perichondrium, is followed by the apoptosis of terminal hypertrophic chondrocytes and the degradation of cartilage matrix; ossification ends with the deposition of bone matrix by osteoblasts on residual calcified cartilage matrix that gives rise to the trabeculae of the primary spongiosa [5–7].

Periosteal bone collar intramembranous ossification precedes the advancing front of endochondral ossification and is carried out by osteoblasts that arise from the mesenchymal cells surrounding the cartilaginous core. Appositional bone growth leads to an increase in diaphyseal diameter due to the deposition of new bone beneath the fibrous layer of the periosteum. The periosteal bone collar extends longitudinally toward both epiphyses, proximally and distally. Bone growth is accompanied by the enlargement of the marrow cavity due to the destruction of bone tissue by osteoclasts [8,9], which dissolve the bone matrix [10,11]. The remodeling of bone matrix by osteoclasts supports the formation of a marrow cavity filled with vessels and hematopoietic cells.

Collagen type XI is a quantitatively minor but essential component of the extracellular matrix [12]. Collagen type XI nucleates the formation and regulates the diameter of heterotypic fibrils [13–15]. Col11a1, Col11a2, and Col2a1 form the triple helical collagen XI in cartilage [16] while alternative combinations are formed in bone, which include the minor fibrillar collagen alpha chains of types V and XI. Minor fibrillar collagens play essential roles in many tissues including heart valve, muscle, tendon, placenta, eye, and skin [17–24].

Structurally, a triple helix is flanked by non-collagenous amino and carboxy terminal domains. Structural diversity arises in the amino terminal domains of the alpha chains of collagen type XI, Col11a1, Col11a2, and Col2a1, due to alternative splicing of the mRNA encoding each of the constituent alpha chains [25–28]. Col2a1 exists in one of two splice variants [29], while numerous splice variants have been reported for Col11a2 [19]. In Col11a1, alternative splicing of exons may generate up to eight possible protein isoforms, which are differentially expressed, both temporally and spatially, during development [30]. Col11a1p6b isoform is restricted to the cartilage periphery underlying the diaphyseal perichondrium during long bone development while the Col11a1p6a78 isoform is associated with early chondrocyte differentiation through pre-chondrogenic mesenchyme and is later restricted to the articular surface [26,30].

The importance of collagen XI in development is evident from the Col11al functional knockout, the chondrodystrophic mouse (cho), which displays an autosomal recessive chondrodysplasia as a result of a point mutation in the Col11a1 gene that causes a reading frame shift and results in a premature stop codon and mRNA instability; a functional knockout of Col11a1 (Col11a1^−/−^) [31,32]. In the absence of Col11a1, an alternate triple helical molecule forms, consisting of Col11a2 and Col5a1, which is unable to compensate for the functional deficiency caused by an absence of Col11a1 [33].

The Col11a1^−/−^ cartilage phenotype was previously characterized with deficiencies in chondrogenesis, epiphyseal cartilage structure, collagen fibrils, cleft palate, and auditory function [34–39]. Here we extend previous analysis of the cartilage phenotype of the Col11a1-deficient mouse and provide information on the mineralized skeleton and bone formation by histology and X-ray microtomography (micro-CT) to specifically assess bone formation in the absence of Col11a1. The data presented here show that Col11a1 depletion resulted in alteration to both trabecular and cortical bone. Characterization of the Col11a1^−/−^ mouse mineralized tissue extends our previous *in vitro* work to further explain the consequences of the loss of Col11a1, influencing osteoblast differentiation and mineralization. These results provide new information on bone development and increase our understanding of human conditions that are caused by mutations in the gene encoding Col11a1, including Stickler syndrome, Marshall syndrome, Wagner syndrome, and fibrochondrogenesis, indicating that Col11a1 plays an essential role in the development of trabecular and cortical bone in addition to the essential role of Col11a1 in cartilage.

## 2. Experimental Section

### 2.1. Mice

The embryos used in this study were housed and euthanized as approved by the Institute of Animal Care and Use Committee of Brigham Young University. All embryos used in this study were at embryonic day 17.5. A total of six wild-type (WT) (+/+) and three homozygous cho (−/−) on a C57Bl6 background were analyzed.

### 2.2. Micro-CT Analysis

Embryos were scanned with a SkyScan 1172 high-resolution micro-CT scanner (Micro Photonics, Aartselaar, Belgium) to generate data sets with a 1.7 μm^3^ isotropic voxel size using an acquisition protocol that consisted of X-ray tube settings of 60 kV and 250 μA, exposure time of 0.147 s, six-frame averaging, a rotation step of 0.300°, and associated scan times were approximately 7 h. Following scanning, a two-dimensional reconstruction stage was used to produce 6000 serial 4000 × 4000 pixel cross-sectional images. Three-dimensional models were reconstructed using a fixed threshold to analyze the mineralized bone phase using ImageVis3D software (Center for Integrative Biomedical Computing, University of Utah, Salt Lake City, UT, USA). A light Gaussian filter (σ = 1.0, kernel = 3) to remove high-frequency noise followed by an adaptive threshold was used to segment the 3D images, which were visually checked to confirm inclusion of complete volume of interest.

Gross geometric measurements were performed using Sky Scan CT Analyzer (CTAn) software (Micro Photonics, Aartselaar, Belgium). Comparisons of shape and cross-sectional area were conducted for long bones, ribs, and spine. CTAn was used to determine trabecular thickness (Tb.Th), trabecular number (Tb.N), trabecular separation (Tb.Sp), degree of anisotropy (DA), and structure model index (SMI) [40–43]. Trabecular thickness, number, and separation measurements were performed on three-dimensional whole bone models of vertebrae, vertebral bodies, and long bones in CTAn. Bone volume (BV) and bone surface (BS) were calculated based on the hexahedral marching cubes volume model of the binarized objects within the volume of interest and the faceted surface of the marching cubes volume model, respectively [43]. Total tissue volume (TV) was defined as the volume-of-interest, which in this case refers to the entire scanned sample. Trabecular bone volume fraction (BV/TV) was calculated from BV and TV values. The degree of anisotropy (DA) and structure model index (SMI) were calculated for long bones. Cross-sectional reconstructions were color-coded according to three density ranges: high-density range (white), intermediate-density range (blue), and low-density range (green).

### 2.3. Trichrome Stain

Embryos were fixed in Bouin’s solution [44] for five days and transferred to 70% ethanol for an additional three days. Ribs and limbs were excised from mice, embedded in paraffin, and sectioned at 6 μm. The sections were stained according to Gomori’s trichrome procedure, where aldehyde fuschin-stained cartilage purple, fast green-stained bone green, and phloxine B-stained blood cells reddish pink [45]. Digital images were obtained with an Olympus BX51 photomicroscope.

### 2.4. Data Analysis

Confidence intervals were determined at 95%. Differences between Col11a1-deficient and WT embryos were identified as those for which the value for the Col11a1-deficient embryo fell outside of the 95% confidence interval for the WT group. Densitometric indices are expressed as mean ± SD.

## 3. Results and Discussion

### 3.1. Changes to Embryonic Skeleton in the Absence of Col11a1 Expression

**Figure 1:**
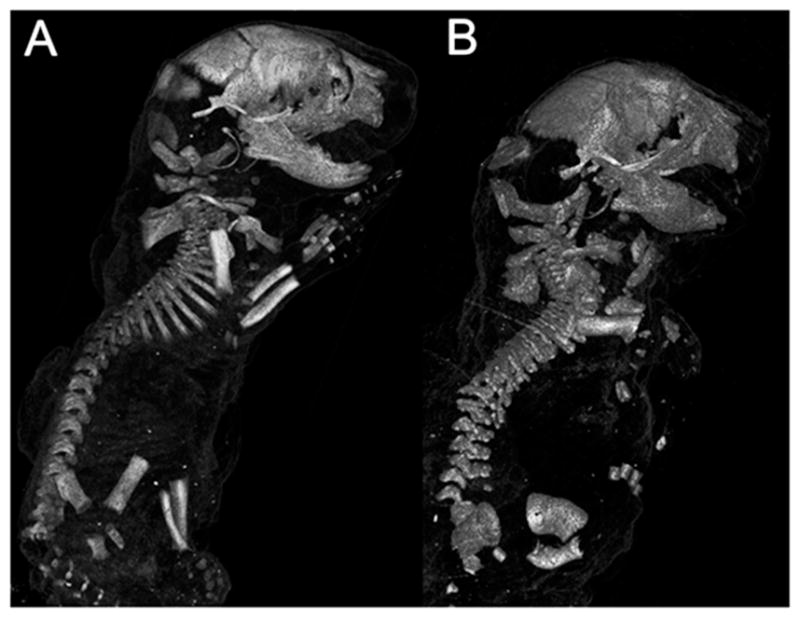
Micro-CT images of whole body for WT and Col11a1-deficient littermates at embryonic day 17.5 (e17.5d). (**A**) WT mouse; and (**B**) Col11a1-deficient mouse. Differences between WT and the Col11a1-deficient mice were consistent with previous characterization, which focused on changes to the cartilage. These images are representative of all WT and mutant mice included in this study.

**Figure 2:**
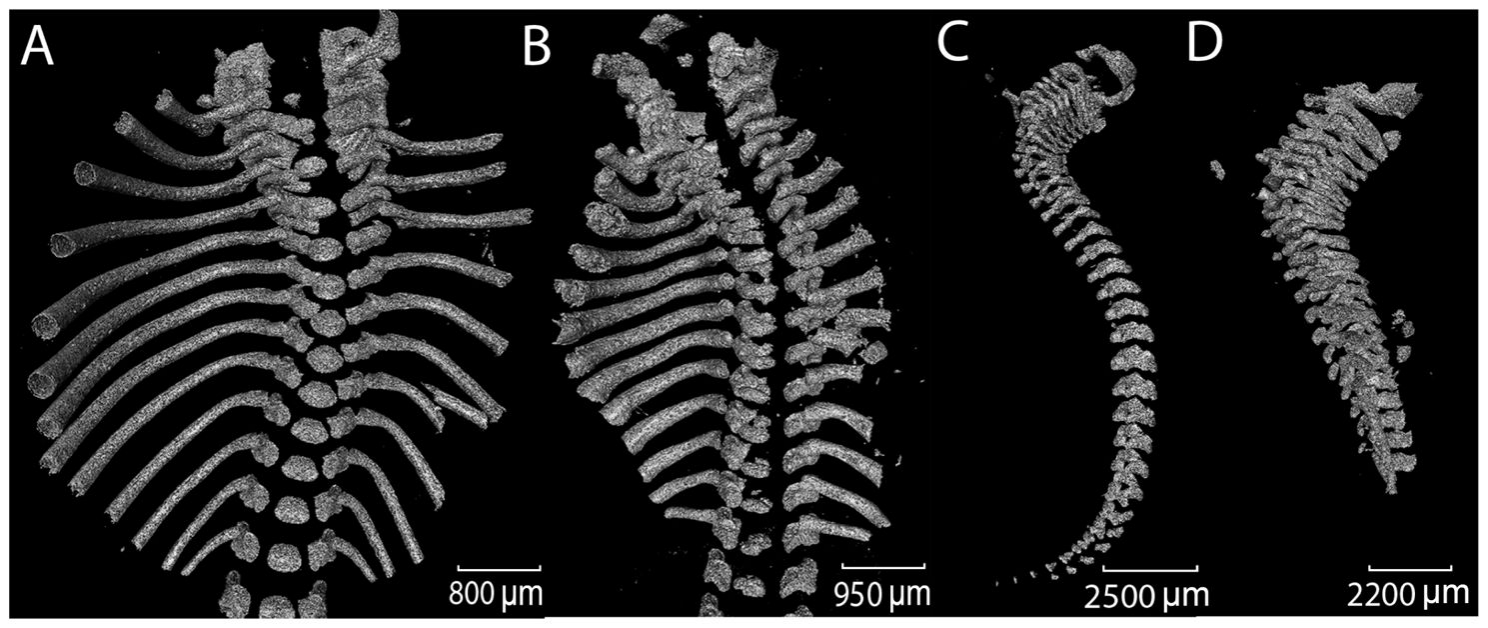
Three-dimensional reconstructions from X-ray micro-CT data of axial skeleton and ribs. (**A** and **C**) WT mouse; and (**B** and **D**) Col11a1-deficient mouse; Differences in spinal curvature and length are apparent upon comparison, as well as a decrease in the separation between vertebrae in the mutant mouse. Lumbar vertebrae from the Col11a1-deficient mouse were less mineralized than the WT mouse and were not visible by micro-CT. These images are representative of all WT and mutant mice included in this study. Scale bar A = 800 μm; scale bar B = 950 μm; scale bar C = 2500 μm; scale bar D = 2200 μm.

Micro-CT data was collected and three-dimensional models of mineralized skeleton were constructed for six WT and three Col11a1-deficient mice. Overall anatomical features observed were consistent with those previously shown [37]. The skeletal deformities characteristic of the Col11a1-deficient mouse included shortened, wider limb bones, shortened snout, small thoracic cage, and shortened spine. These were apparent in the three-dimensional reconstructions of mineralized skeleton (Figure 1). To analyze the shortened spine and vertebrae in more detail, a reconstruction of the spine and ribs was made and is shown in Figure 2. Analysis of three-dimensional reconstructions from X-ray micro-CT data revealed a decrease in separation between the vertebrae and an increase in the height of individual vertebrae in the Col11a1-deficient mice compared to WT littermates. The extent of mineralization was reduced in the lower thoracic and lumbar vertebrae in the absence of Col11a1. Mineralization of the lumbar vertebrae from the Col11a1-deficient mice was below the limit of detection and, therefore, was not visible in Figure 2.

### 3.2. Analysis of Vertebrae

**Figure 3:**
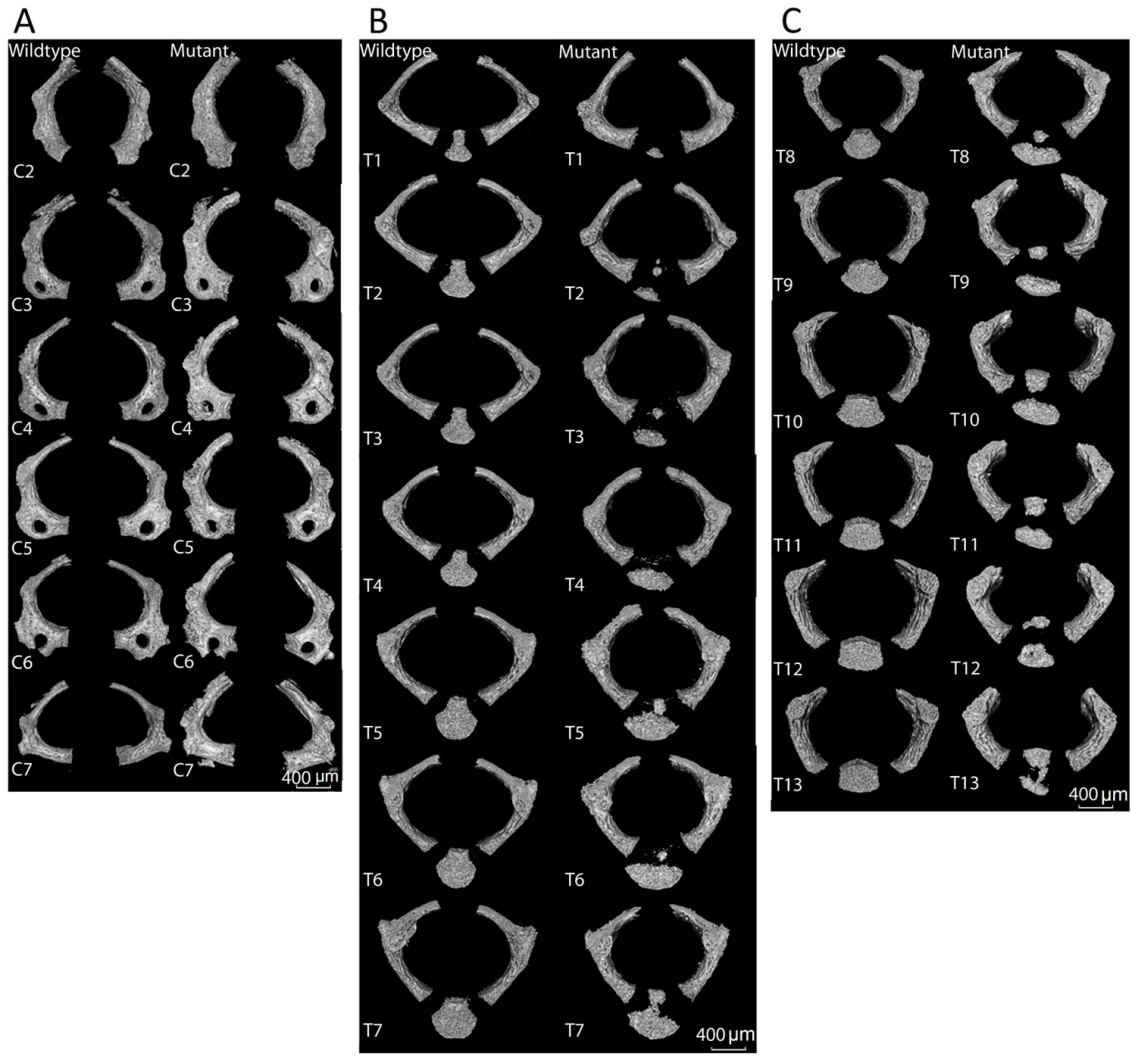
Pairwise comparison of shape and size of individual vertebrae. (**A**) Cervical vertebrae C2 through C7. (**B**) Thoracic vertebrae T1 through T7. (**C**) Thoracic vertebrae T8 through T13. Differences in shape and surface characteristics were apparent. Vertebral bodies of T8–T11 were less mineralized in the Col11a1-deficient mouse compared to WT. Vertebral bodies in WT mouse in comparison to Col11a1-deficient mouse show evidence of hemi-vertebrae malformation with decreased mineralization along the midline of the vertebra. Scale bar A–C = 400 μm.

The gross morphology of each vertebra was compared among littermates. In the absence of Col11a1, the vertebral arches exhibited a more rounded shape, in contrast to the ovoid shape of the vertebrae from control mice (Figure 3). Vertebral bodies of the thoracic and lumbar regions in the Col11a1-deficient mice were reduced in size, and appeared to have an altered shape and incomplete mineralization. Further, in contrast to WT, which exhibited a single mineralized component that comprised the vertebral body, multiple smaller mineralization foci and a lack of mineralization along the midline of the vertebral bodies was observed in the Col11a1-deficient mice. The morphological changes in vertebral body formation were consistent with changes that lead to congenital spinal deformities, which contribute to scoliosis and kyphosis [46].

### 3.3. Bone Microarchitectural Parameters Dependent upon the Expression of Col11a1

**Figure 4:**
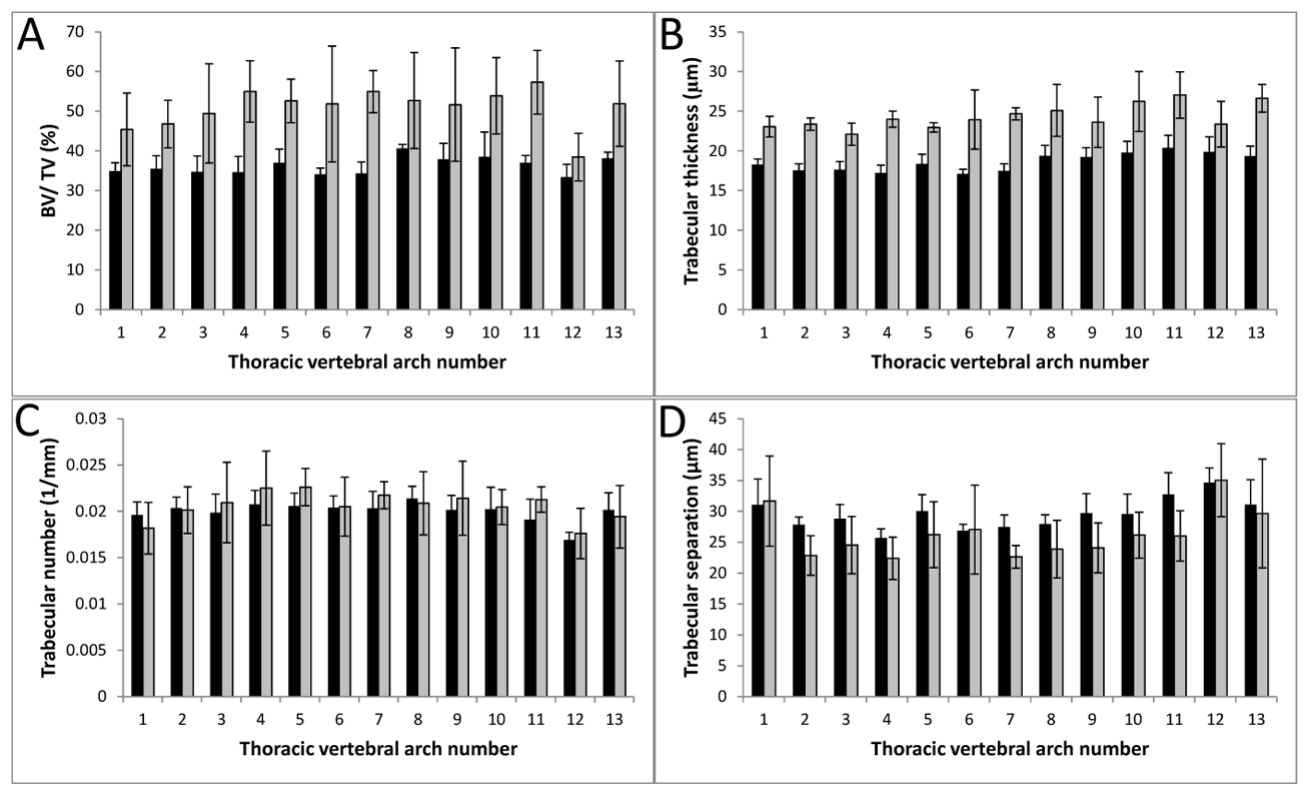
Densitometric analysis of vertebral arch mineralization from the thoracic region. (**A**) Percent bone volume (BV/TV) was determined for each thoracic vertebral arch and values were averaged among six wild-type and three Col11a1^−/−^ e17.5d embryos. Col11a1^−/−^ (grey) vertebral arches had a greater percent bone volume consistently comparer to wild-type littermates (black). (**B**) Trabecular thickness (μm) was determined for each thoracic vertebral arch and values were averaged among six wild-type and three Col11a1^−/−^ e17.5d embryos. Trabecular thickness was consistently greater in the Col11a1^−/−^ embryos compared to wild-type littermates. (**C**) Trabecular number (1/mm) was determined for each thoracic vertebral arch and values were averaged among six wild-type and three Col11a1^−/−^ e17.5d embryos. No significant difference in trabecular number was observed between wild-type and Col11a1-deficient embryos. (**D**) Trabecular separation (μm) was determined for each thoracic vertebral arch and values were averaged among six wild-type and three Col11a1^−/−^ e17.5d embryos. No significant difference in trabecular number was observed between wild-type and Col11a1-deficient embryos. Error bars represent mean ± SD. Average values of all differences are presented in Table 1.

**Figure 5:**
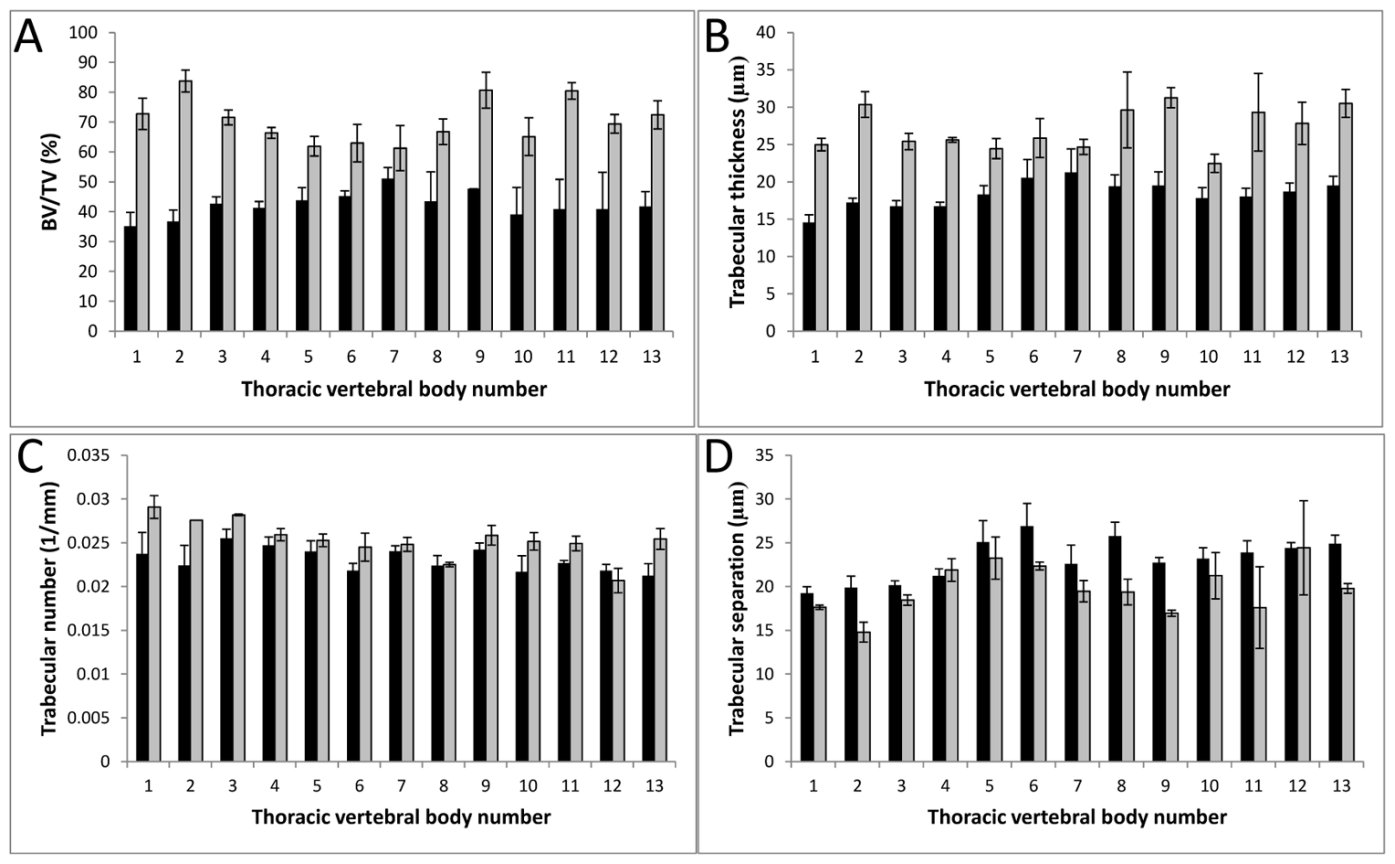
Densitometric analysis of the vertebral bodies from the thoracic region. **(A)** Percent bone volume (BV/TV) was determined for each thoracic vertebral body and values were averaged among six wild-type and three Col11a1^−/−^ e17.5d embryos. Col11a1^−/−^ (grey) vertebral bodies had a greater percent bone volume consistently comparer to wild-type littermates (black). (**B**) Trabecular thickness (μm) was determined for each thoracic vertebral body and values were averaged among six wild-type and three Col11a1^−/−^ e17.5d embryos. Trabecular thickness was consistently greater in the Col11a1^−/−^ embryos compared to wild-type littermates. (**C**) Trabecular number (1/mm) was determined for each thoracic vertebral body and values were averaged among six wild-type and three Col11a1^−/−^ e17.5d embryos. No significant difference in trabecular number was observed between wild-type and Col11a1-deficient embryos. (**D**) Trabecular separation (μm) was determined for each thoracic vertebral body and values were averaged among six wild-type and three Col11a1^−/−^ e17.5d embryos. While no significant difference in trabecular separation was observed between wild-type and Col11a1-deficient embryos for many of the individual vertebral bodies, there are some for which a significant decrease was detected. Error bars represent ± SD. Average values of all differences are presented in Table 2.

Quantitative changes to bone density of the vertebrae T1–T13 were identified in the Col11a1-deficient mice compared to WT littermates (Figures 4 and 5). Microarchitectural parameters were determined for the thoracic vertebral arches and bodies, T1–T13 (Tables 1 and 2); indices describing trabecular thickness, (Tb.Th), trabecular number, (Tb.N), trabecular separation (Tb.S), and trabecular percent bone volume (BV/TV) were determined. In the vertebral arches, the trabecular thickness and percent bone volume were greater in the Col11a1-deficient mice compared to WT littermates (31.7% and 32.8% increase, respectively). While trabecular spacing and number of Col11a1-deficient mice showed differences when compared to WT, these differences were small and not statistically significant. Trabecular thickness and percent bone volume were greater in the vertebral bodies of the Col11a1-deficient mice compared to WT littermates (80.4% and 67.2% increase, respectively) and trabecular spacing decreased in the Col11a1-deficient mice compared to WT littermates (a decrease of 17%). As with the vertebral arches, a difference in trabecular number was observed, but the difference was not significant.

### 3.4. Col11a1-dependent Changes in the Ribs

**Figure 6:**
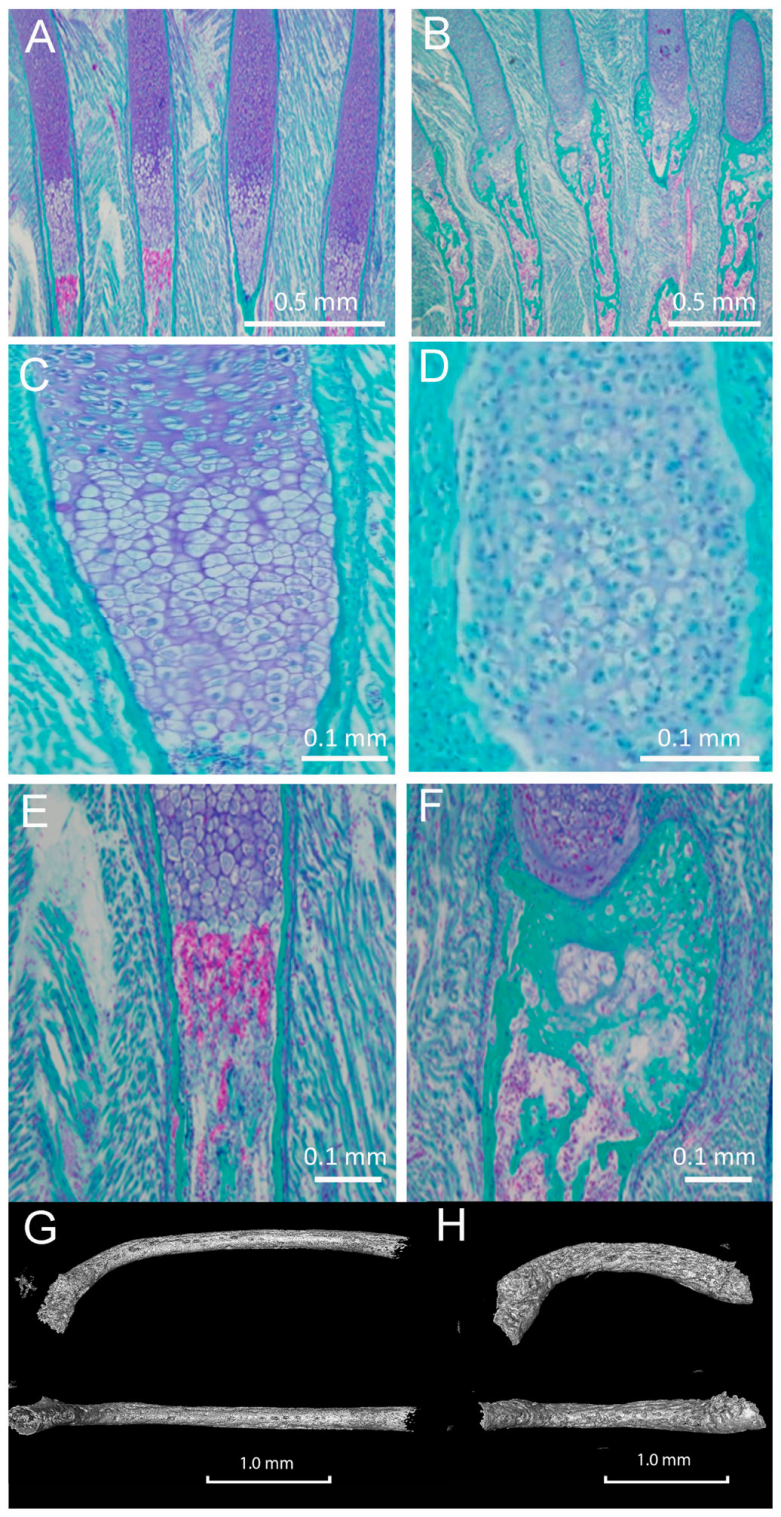
Comparison of ribs between WT and Col11a1-deficient mice. (A, C, E, G); WT (B, D, F, H); Col11a1-deficient mouse. (A and B); Histological differences in four adjacent ribs for WT and Col11a1-deficient mice. Trichrome staining rendered mineralized tissue green, cartilage tissue deep blue, and blood cells pink/purple. (C and D); Histological differences within proliferative/hypertrophic chondrocytes from WT and Col11a1^−/−^. (E and F); Col11a1-deficiency led to an increase in mineralization in the newly-formed bone collar in the Col11a1-deficient mouse compared to WT. Images in C, D, E, F are from representative ribs not appearing in A and B. (G and H); Reduced length, increased curvature of the ribs was apparent in the Col11a1-deficient mice compared to WT. Proximal is oriented to the left for each rib, with the distal growth plate located on the right. Scale bars A and B = 0.5 mm; C and D = 0.1 mm; E and F = 1.0 mm.

In the absence of Col11a1, ribs developed a more severe curvature at the proximal end, near the point of attachment of the head and tubercle of the rib to the costal demifacet and transverse costal facet of the vertebrae respectively, apparent in Figures 1 and 2. Histologic sections demonstrated an increase in mineralization in the ribs of Col11a1-deficient mice compared to WT controls (Figure 6). The ribs of Col11a1-deficient mice were shorter and thicker than WT controls (Figure 6).

### 3.5. Histological Analysis of Embryonic Long Bone Formation

**Figure 7:**
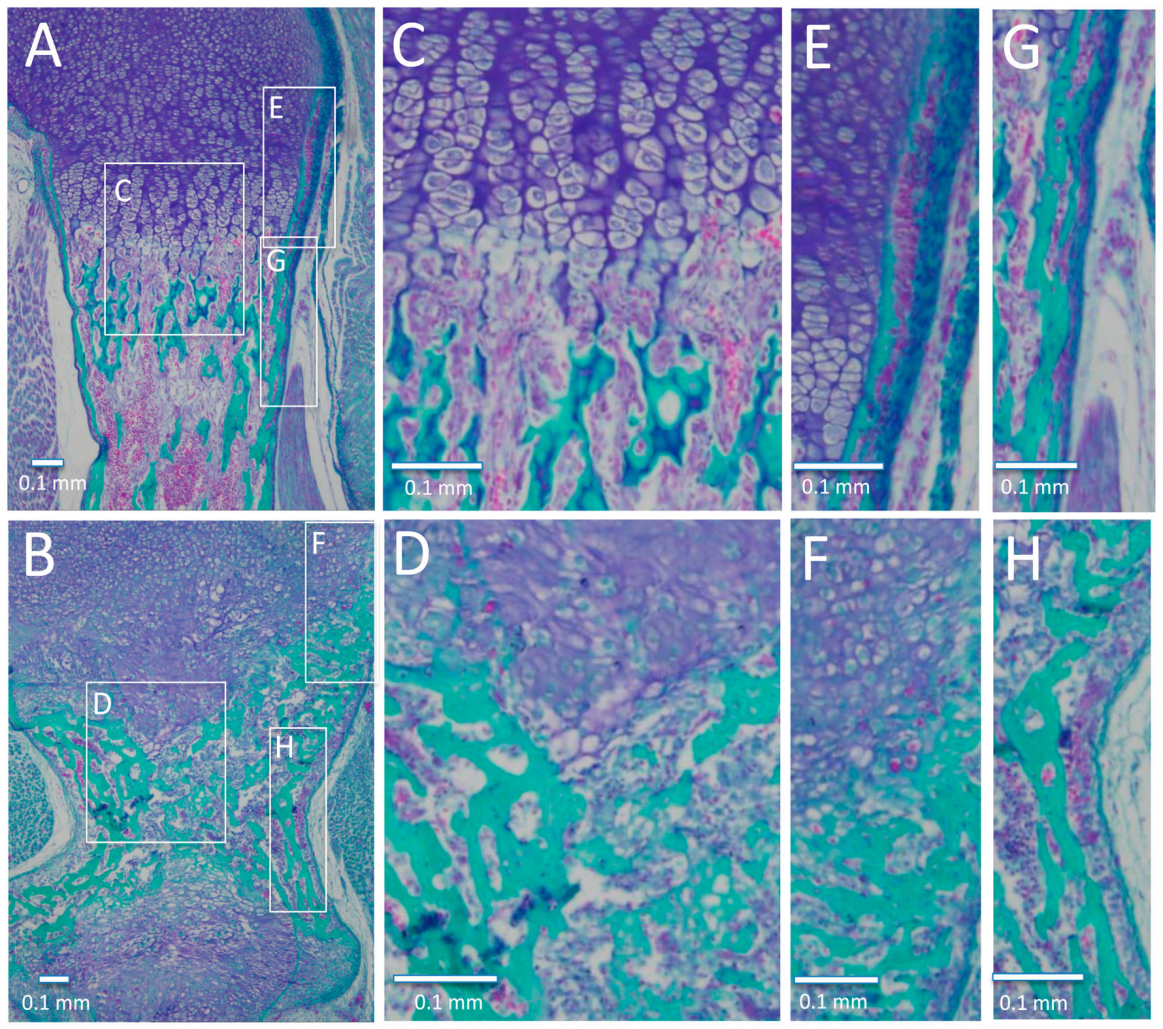
Histological differences in the humeri of WT and Col11a1-deficient mice. (**A**, **C**, **E**, **G**); WT; (**B**, **D**, **F**, **H**); Col11a1-deficient mice. Trichrome staining was used to identify mineralized tissue (green), compared to cartilage tissue (blue). (**A** and **B**); Upper and lower hypertrophic and mineralized zone, with diaphysis and both growth plates shown for the Col11a1-deficient humerus. (**C** and **D**); Transition from hypertrophic to mineralized zone demonstrating altered hypertrophic cell size, a more abrupt transition from hypertrophic cartilage to mineralized tissue, and altered bone deposition. (**E** and **F**); Newly formed bone collar adjacent to the upper hypertrophic region demonstrates a thicker mineralized region adjacent to the cartilage in the mutant compared to WT. (**G** and **H**); Bone collar near the diaphysis from WT and Col11a1-deficient mice. Scale bars = 0.1 mm.

Trichrome staining was used to analyze mineralization in the long bones including femur, tibia, humerus, radius, and ulna of WT and Col11a1-deficient mice. Figure 7 demonstrates histological differences in the humerus. An increase in mineralized tissue was observed immediately adjacent to the lower hypertrophic zone of the growth plates (compare Figure 7A to 7B, and 7C to 7D). An increase in mineralized tissue was also observed at the periosteal surface of the newly formed bone collar, although the intensity of fast green staining for mineralized tissue was lower than that observed in the WT mice (compare Figure 7E to 7F for newly formed bone collar in the upper hypertrophic region and 7G to 7H for bone collar near the diaphysis). Analysis of this data indicated a defect in perichondrial bone formation in the absence of Col11a1.

### 3.6. Metaphyses, Diaphysis, and Cross-Sectional Area of the Col11a1-Deficient Forelimbs

**Figure 8:**
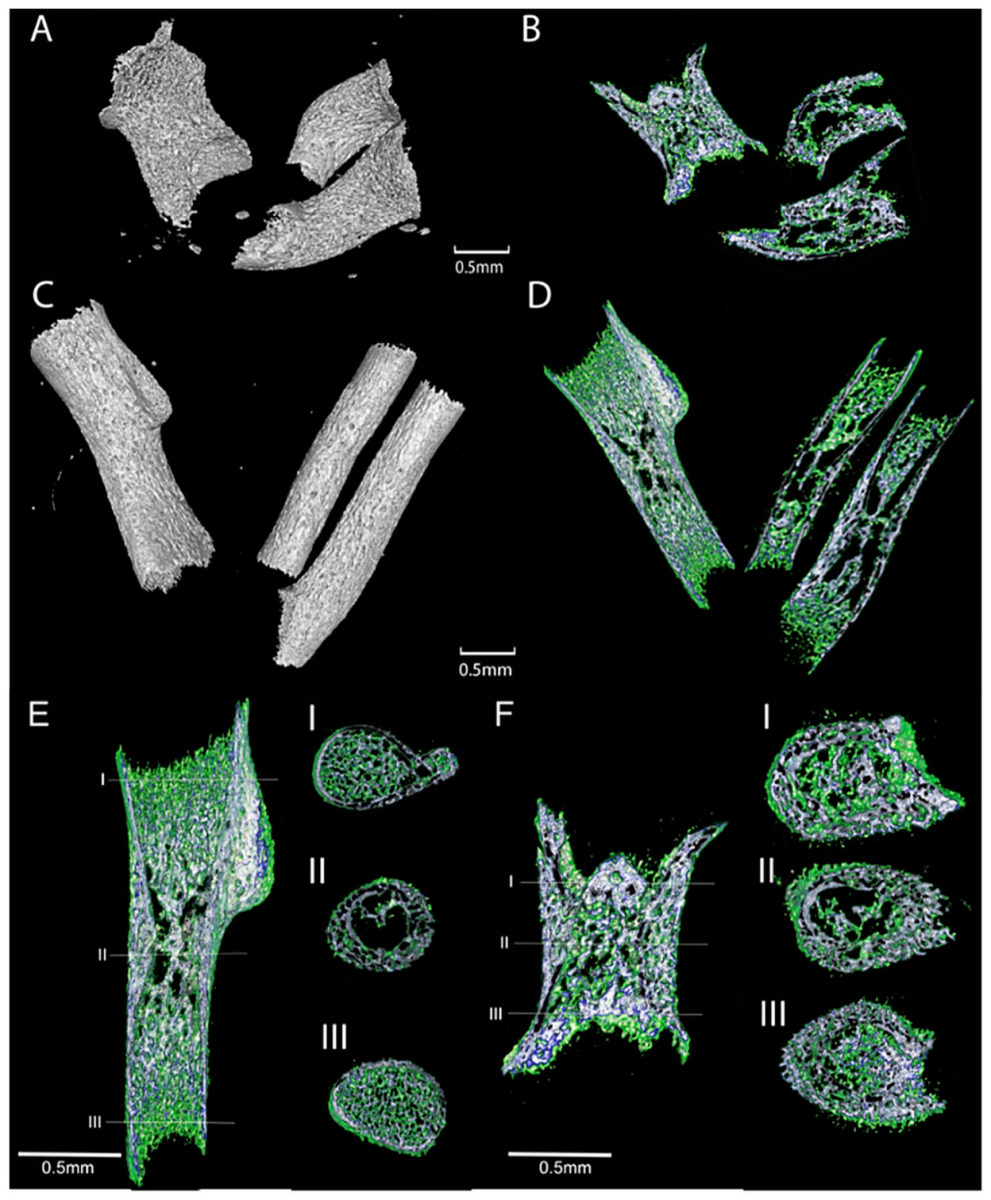
X-ray micro-CT images of forelimbs. (**A** and **B**); Col11a1-deficient mouse. (**C** and **D**); WT mouse; (**A**) Radius, ulna, and humerus of Col11a1-deficient mouse. The mineralized portions of the bones were shorter and wider in the Col11a1-deficient mice compared to (**C**) WT littermates (Table 3). Deltoid tuberosity was apparent in the WT humerus but absent in the Col11a1-deficient mouse. (**B** and **D**); Longitudinal cross-sections of each forelimb, shown in A and C, respectively. Mineralized tissue was assigned a color dependent upon three density ranges: low-density range (green), intermediate-density (blue), and high-density (white). Marrow space within the Col11a1-deficient limb showed regions of higher bone density near the proximal growth plates and those of very low density near the distal growth plates when compared to analogous regions in the WT littermate. (**E** and **F**); Cross-section of humeri at diaphysis, distal, and proximal metaphyses. (**E**) WT (**F**) Col11a1-deficient mouse. Col11a1-deficient humerus was wider and more cylindrical than WT. Mineralized tissue was assigned a color dependent upon three density ranges: low-density range (green), intermediate-density (blue), and high-density (white). Trabecular bone was denser in Col11a1-deficient mice compared to WT at proximal metaphysis. Trabecular bone is less dense in Col11a1-deficient mice compared to WT at distal metaphysis. Bone collar is less dense but thicker in Col11a1-deficient mice compared to WT littermate. Scale bars = 0.5 mm.

The mineralized portion of long bones from Col11a1-deficient mice were an average of 41% shorter than the WT humerus and femur (Figure 8). The Col11a1-deficient mice humeri exhibited an abnormally cylindrical shape atypical of a normal developing humerus, and lacked the deltoid tuberosity seen in the WT littermates (Figure 8). The bones of the Col11a1-deficient mice appeared wider at all points along the length of the bone (Figure 8) and on average were 24% wider at the diaphysis, 15% wider at the proximal metaphysis, and 47% wider at the distal metaphysis (Table 3). Average cross-sectional area was found to be 80% greater at the diaphysis, 56% greater at the proximal metaphysis, and 26% greater at the distal metaphysis in the absence of Col11a1. Interestingly, the Col11a1-deficient long bones displayed an increase in mineralized tissue at the proximal metaphysis and a decrease of mineralized trabecular bone at the distal metaphysis.

Trabecular thickness, trabecular separation and trabecular percent bone volume were increased in the forelimb bones of the Col11a1-deficient mice. Analysis of microarchitectural indices at the proximal metaphysis of the humerus showed differences in trabecular thickness (93% increase in Tb.Th), trabecular separation (17% increase in Tb.Sp), and trabecular percent bone volume (73% increase in BV/TV) in the absence of Col11a1 expression. While consistently decreased in samples, the difference in trabecular number did not fall outside the 95% confidence interval for WT values (Table 4). No significant difference was detected for isotropy values or structure model index indicating similar relative prevalence of rods and plates in the three-dimensional structure of the trabecular bone for WT and Col11a1-deficient mice (Table 4).

## 4. Conclusions

Three-dimensional models were created from X-ray micro-CT images of skeletons from Col11a1-deficient mice and these were compared to WT littermates. Relative to WT littermates, the percent bone volume was increased in the absence of Col11a1 gene expression. Trabecular thickness and number were increased while trabecular separation was decreased in the Col11a1-deficient mice. This study provides quantitative information on the microarchitecture of the skeleton and the role that Col11a1 plays in bone development.

Differences in skeletal development were observed in the deltoid tuberosity of the humerus. The deltoid tuberosity was not formed in the absence of Col11a1 expression. Periosteal bone thickness was greater in the absence of Col11a1 expression compared to WT littermates, and this increase in bone thickness may be due to excessive appositional growth and mineralization within the periosteum, resulting in an increase in radial growth at the perichondrium relative to that of the control littermates. This finding may indicate a lack of regulation in bone collar formation in the absence of the Col11a1 gene product and may indicate that Col11a1 plays an essential role in the formation of the bone collar.

While the function of Col11a1 is best characterized in the context of cartilage, Col11a1 is also expressed in many other tissues, including bone. Recently, a role for Col11a1 in osteoblast function was identified in a study in which osteoblast maturation was accelerated in the absence of specific Col11a1 isoforms and inhibited in the presence of a recombinant fragment of Col11a1 [47]. Thus, recent findings indicate a direct role in osteoblast function and differentiation, which is distinct from the previously reported role in the assembly of the extracellular matrix synthesized by chondrocytes.

Phenotypic overlap between the Col11a1 mutation and that of other structural molecules of the extracellular matrix may indicate a shared function or a direct molecular interaction between the two constituents within the matrix. Candidate molecules for which a phenotypic overlap with Col11a1 exists include Col2a1, link protein, chondroitin sulfate sulfotransferase 1, PTHrP, Indian hedgehog, and FGFRs [48–52]. Mice overexpressing BMP4 in cartilage have widened bones containing thick trabeculae, possibly because of expansion of cartilage anlagen [53]. Thickened trabeculae were also observed in a Col11a2-BMP4 transgenic mouse at 18.5 days of embryonic development. In the Col11a2-BMP4 mouse, the epiphyseal cartilage of the humeri were widened compared to WT. Additionally, the diaphyses undergoing mineralization were also widened, accompanied by the observation of thickened trabecular bone in the marrow cavities. When Noggin expression was placed under the control of the Col1a1 promoter in transgenic mice, micro-CT analysis revealed a greater volume of trabecular bone during embryonic stage 17.5 days to three weeks after birth, when compared to WT [53].

It is possible that the changes in bone microarchitecture observed in the absence of the Col11a1 gene product may be explained by primary changes to the structure of the cartilage anlagen during endochondral ossification, leading to subsequent changes in bone microarchitecture secondarily [54]. A wider cartilaginous anlagen may result in the production of a widened bone structure. Additionally, altered properties of the cartilaginous anlagen due to the absence of Col11a1 may result in changes to distribution and delivery of cell signaling molecules that control bone growth and the spatial and temporal control of bone mineralization. Future studies are needed to focus on potential mechanisms of Col11a1’s effect on mineralization, directly and indirectly.

Mutations in the genes encoding collagen type XI alpha chains result in a number of spondylo-epiphyseal dysplasias [48]. Among these conditions, are the human chondrodysplasias, Stickler syndrome, Marshall syndrome, Wagner syndrome, and fibrochondrogenesis [49,55,56]. Collagen type XI-related syndromes present a number of clinical skeletal symptoms, including abnormal epiphyseal development, irregularity of the margins of the vertebral bodies, thick calvaria, short stature, and intracranial calcifications (OMIM: 154780, 108300, 143200).

Overall, the changes observed in this study suggest that the absence of Col11a1 gene expression in developing bone resulted in thickened trabecular bone and reduction in endosteal bone turnover, contributing to alterations in marrow cavity formation and an increase in periosteal bone apposition leading to a defect in primary spongiosa formation and a thicker bone collar. These data suggest that Col11a1 may be a regulator of osteogenesis and mineralization of the skeleton during endochondral ossification. The changes to the bone collar observed in these studies suggest a role for Col11a1 in intramembranous bone formation. Future investigations from our laboratory will focus on determining the molecular mechanism of Col11a1 involvement in chondrogenic and osteoblastic differentiation during endochondral and intramembranous ossification.

The impact of a Col11a1-deficiency on the formation of vertebral bodies was an unexpected result. A review of the literature indicated that hemivertebrae formation can be associated with two different types of defects, one that occurs during the prechondral stage of vertebral body formation and one that occurs at the ossification stage. It is interesting to note that Col11a1 mutations have been identified by genome-wide association studies for lower back pain and lumbar disc degeneration in some populations [57].

## Figures and Tables

**Table 1:** Densitometric indices for the vertebral arches (mean % difference ± SD) between WT and Col11a1^−/−^.

	BV/TV (%)	Tb.Th (μm)	Tb.N (1/mm)	Tb.Sp (μm)
% difference	+31.7	+32.8	−1.01	+0.03
SD	3.9	3.4	1.0	1.0
	*p* < 0.05	*p* < 0.05	ns	ns

BV/TV, Tb.Th, Tb.N and Tb.Sp are reported as percent difference between Col11a1-deficient mice compared to WT littermates, reported as mean with SD. BV/TV (%) increased for Col11a1^−/−^ compared to WT. Statistical differences are reported as p values unless determined to be not significant (ns). Control mice (n = 6), Col11a1-deficient mice (n = 3).

**Table 2:** Densitometric indices for the vertebral bodies (mean % difference ± SD) between WT and Col11a1^−/−^

	BV/TV (%)	Tb.Th (μm)	Tb.N (1/mm)	Tb.Sp (μm)
% difference	+80.4	+67.2	+8.26	−17
SD	8.5	7.9	1.0	2.0
	*p* < 0.05	*p* < 0.05	ns	*p* < 0.05

BV/TV, Tb.Th, Tb.N and Tb.Sp are reported as percent difference between Col11a1-deficient mice compared to WT littermates, reported as mean with SD. BV/TV (%), Tb.Th and Tb.N increased while Tb.Sp decreased in Col11a1^−/−^ compared to WT. Statistical differences are reported as p values unless determined to be not significant (ns). Control mice (n = 6), Col11a1-deficient mice (n = 3).

**Table 3:** Structural indices for humeri are reported as mean + SD for WT and Col11a1^−/−^.

Genotype	Length (μm)	Proximal Metaphysis Diameter (μm)	Diaphysis Diameter (μm)	Distal Metaphysis Diameter (μm)
WT	2409 ± 33.6	806 ± 16.9	592 ± 11.6	528 ± 15.2
Col11a1^−/−^	1422 ± 65.5	930 ± 38.4	735 ± 37.4	778 ± 86.1
% difference	41.0	15.4	24.2	47.4
	*p* < 0.0001	*p* < 0.05	*p* < 0.05	*p* < 0.05

Values are reported as mean ± SD. Statistical differences are reported as p values unless determined to be not significant (ns). Control mice (n = 6), Col11a1-deficient mice (n = 3).

**Table 4:** Densitometric indices for the humeri (mean + SD) WT and Col11a1^−/−^.

Genotype	BV/TV (%)	SMI	DA	Tb.Th (μm)	Tb.N (1/mm)	Tb.Sp (μm)
WT	25.3 ± 2.9	2.1 ± 0.17	0.90 ± 0.025	18.32 ± 0.22	13.8 ± 1.5	33.2 ± 1.81
Col11a1^−/−^	43.7 ± 4.1	1.87 ± 0.033	0.84 ± 0.082	35.3 ± 3.7	12.4 ± 0.81	38.9 ± 1.19
% difference	72.7	11.0	0.1	91.6	10.2	17.2
	*p* < 0.05	ns	ns	*p* < 0.05	ns	*p* < 0.05

BV/TV, Tb.Th, Tb.N, and Tb.Sp, are reported as mean ± SD. Statistical differences are reported as p values unless determined to be not significant (ns). Isotropy values (DA) range from 0 (total isotropy) to 1 (total anisotropy). Structure model index (SMI) indicating relative prevalence of rods and plates in three-dimensional structure range from 0 (plate-like) to 3 (rod-like). Control mice (n = 6), Col11a1-deficient mice (n = 3).
